# Demographic and clinical profile of patients undergoing colonoscopy at a tertiary care center in Nepal: a retrospective cross-sectional study

**DOI:** 10.1097/MS9.0000000000002003

**Published:** 2024-04-09

**Authors:** Pasanda Sharma, Prakash Sapkota, Ram B. Gurung, Prabhat Silwal, Binay Yadav, Nishchal Gupta, Shikha Pathak, Sahasra Joshi, Yadvinder Singh

**Affiliations:** a Deparment of Internal Medicine; b Dhulikhel Hospital, Kathmandu University School of Medical Sciences, Dhulikhel, Kavre, Nepal

**Keywords:** colonoscopy, colorectal carcinoma, colorectal polyp, cross-sectional study

## Abstract

**Background:**

Colonoscopy is widely used as a tool for diagnosis, screening and treatment of various pathologies in the rectum, colon, and terminal ileum. The aim of this study is to evaluate the demographic, clinical, and histological parameters of the records of patients undergoing colonoscopy at a tertiary care hospital in Nepal.

**Materials and methods:**

This retrospective cross-sectional study included the hospital records of all patients who underwent colonoscopy from 2015 to 2019 in a tertiary care centre in Nepal.

**Results:**

A total of 1255 records were included in the study. The mean and standard deviation of age were 43.8 and 19 years, respectively. Among the total study population, 61.9% were males and 38.1% were females. Common indications for colonoscopy included lower gastrointestinal bleeding (27.7%), altered bowel habit (26.9%) and persistent or recurrent abdominal pain (17.3%). Only 3% of the patients who underwent colonoscopy had inadequate bowel preparation. The overall diagnostic yield of colonoscopy was 57.5%. Findings during colonoscopy included colorectal polyp (19.4%), internal haemorrhoids (8.2%) and colitis (6.5%). Having an age of 50 or more years was significantly associated with the presence of colorectal adenocarcinoma (*P*=0.017, χ^2^ test) with an odds ratio of 2.35 (95% CI: 1.14, 4.89). Furthermore, having a female sex was found to be significantly associated with the presence of colorectal adenocarcinoma (*P*=0.012, χ^2^ test) with an odds ratio of 2.43 (95% CI: 1.19, 4.97).

**Conclusion:**

In the authors’ study, a smaller proportion of the colonoscopies was aimed at screening for colorectal carcinoma (CRC), when compared to studies in developed countries. The sex predisposition of CRC in the authors’ study is in contrast to the global trend. The authors recommend conducting studies to determine the risk factors and need for CRC screening in the Nepalese population.

## Introduction

Highlights

- At our centre, common indications for colonoscopy were lower gastrointestinal bleeding, altered bowel habits and persistent abdominal pain.
- Common findings during the colonoscopy included colorectal polyp, internal haemorrhoids and colitis
- Only 3% of the patients who underwent colonoscopy had inadequate bowel preparation.
- The overall diagnostic yield of colonoscopy was 57.5%. Polyp detection rate was 19.4%.
- Among the patients who underwent colonoscopy at our centre, having an age of 50 or more years as well as being female were both significantly associated with the presence of colorectal adenocarcinoma with odds ratios of 2.35 and 2.43, respectively.

Colonoscopy is used in the diagnosis, screening, and treatment of various abnormalities in the rectum, colon, and terminal ileum. It is regarded as the gold standard procedure in the evaluation of patients with lower gastrointestinal symptoms such as abdominal pain, diarrhoea, hematochezia, difficulty defecation, and mucoid stool^1^. It can detect colonic polyps, ulcers, bleeding, inflammation, and tumours^2^. It is also regarded as the best modality for colon cancer screening and surveillance^2^. As a therapeutic tool, it allows haemostasis by endoscopic clipping, relief of colonic obstruction by endoscopic balloon dilation or colonic stenting, and polypectomy for colorectal polyps^2^.

In 1966, the first complete flexible endoscopic examination of the large intestine was possible by tying a gastroscope to the rectal end of a long ingested string^3^. Today, colonoscopy is performed through a long flexible tube called a colonoscope containing a fibre-optic camera at its tip that can enter through the patient’s rectum^3^. Colonoscopy has evolved from a procedure that requires hospitalization to one that can be performed in an outpatient office setting^3^.

Despite the importance of colonoscopy in clinical practice, only a few studies have described patients undergoing colonoscopy in Nepal^4–7^. In this study, we aim to evaluate the demographic characteristics, clinical presentation, colonoscopy observations and histological findings of patients undergoing colonoscopy by analyzing the records at a tertiary care hospital in Nepal.

## Materials and methods

### Study design and setting

This was a retrospective cross-sectional study of hospital records of all patients who underwent colonoscopy from 1 January 2015 to 31 December 2019 in the Unit of Gastroenterology at a tertiary care centre in Nepal. This study was done in accordance with the Strengthening the reporting of cohort, cross-sectional and case-control studies in surgery (STROCSS) 2021 guidelines^8^. Ethical approval was obtained from the Institutional Review Committee (IRC) of the same institute. The study protocol conforms to the ethical guidelines of the Declaration of Helsinki as reflected in approval by the institution’s IRC.

### Procedure of colonoscopy

All patients were instructed to follow a liquid diet on the day before the colonoscopy. Patients were advised to then take a 10 mg bisacodyl tablet orally in the evening and fast overnight. On the day of colonoscopy, 2 l of bowel preparation solution was consumed by each patient. The solution contained 118 gm of polyethylene glycol 4000, 2.93 gm of sodium chloride, 1.48 gm of potassium chloride, 3.37 gm of sodium bicarbonate and 11.36 gm of anhydrous sodium sulfate mixed in 2 l of drinking water. The patients were advised about bowel preparation by trained nurses and provided with printed instructions. Colonoscopy was performed after patients had 8–10 episodes of loose stool.

Inadequate bowel preparation was reported if the patient did not follow these instructions for bowel preparation or mucosa inadequately visualized on colonoscopy.

All colonoscopies were performed by using Olympus CF-Q145L, CF-Q165I or CF-Q165L video colonoscopes.

When required, tissue samples from lesions were taken and sent for histopathological analysis.

### Study participants and selection criteria

**Figure 1:**
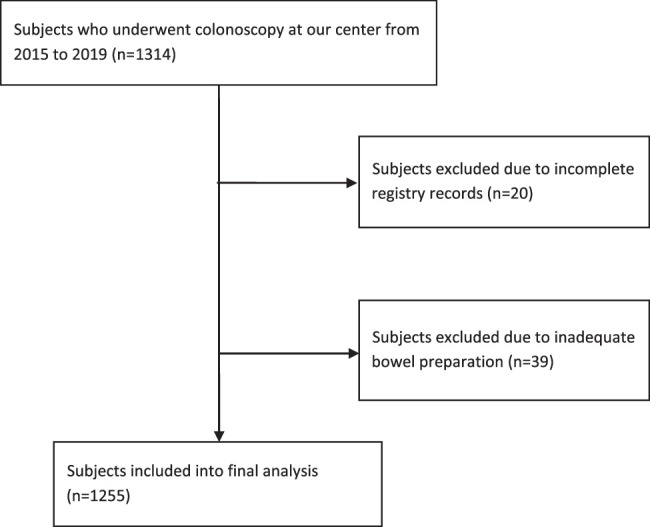
Flowchart showing participant’s inclusion and exclusion from this cross-sectional study.

Records of all patients who underwent colonoscopy at our centre during the study period amounted to 1314 records. We excluded records with incomplete data (*n*=20, 1.5%) and records of patients who had inadequate bowel preparation at the time of colonoscopy (*n*=39, 3%). Hence, records of 1255 patients were included in the final analysis (Fig. 1).

### Data collection

Entries of patients in the physical colonoscopy register present in the Unit of Gastroenterology were reviewed and relevant data were retrieved and entered into a spreadsheet in IBM Statistical Package for Social Sciences (IBM SPSS Statistics for Windows, Version 27.0, IBM Corp). The confidentiality and anonymity of the participants were maintained and no personal or identifiable data were collected when retrieving the data from the register. The register is maintained by dedicated staff who had no further involvement in the study.

### Study variables

The variables studied included age, sex, indications for colonoscopy, findings of colonoscopy and histopathological findings.

### Study size

We considered the presence of adenocarcinoma as the primary outcome. The sample size was calculated based on the standard formula^9^ for cross-sectional studies *n*=Z^2^_a/2_*P*(1−P)/d^2^, where Z_a/2_ was 1.96 for 95% level of confidence, P is the estimated proportion of prevalence of primary outcome and d is the desired level of precision which was set at 0.02. The prevalence proportion (P) was considered to be 6.48%^4^, and thus, the total sample size study required was 642, including an additional 10% to avoid errors. A convenience sampling method was implemented to select participants. However, all patients who fulfilled the study criteria were included in the study, so the final sample size was 1255.

### Statistical analysis

Continuous variables were described in terms of mean, 95% CI, standard deviation (SD) and range, whereas categorical variables were reported as frequencies and percentages. Pearson’s χ^2^ test was applied to assess the significance of the association between two categorical variables with a *P* value of less than 0.05 considered statistically significant. Odds ratio (OR) was used to study the strength of associations between the categorical variables. All statistical analyses were performed using the Statistical Package for Social Sciences (IBM SPSS Statistics for Windows, Version 27.0, IBM Corp).

## Results

### Demographic characteristics

**Figure 2:**
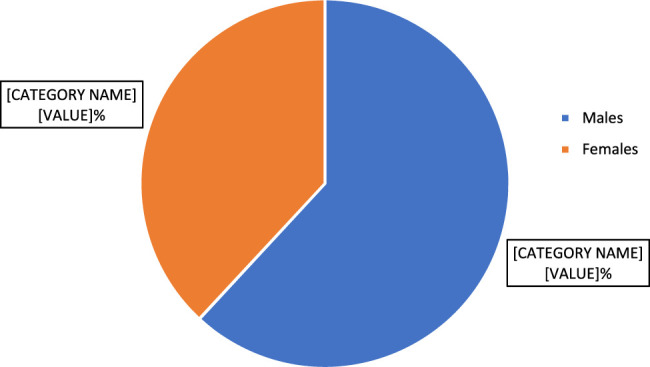
Gender distribution of the study participants (*n*=1255).

A total of 1255 patients were included in this study. The mean age of the patients was 43.8 years (95% CI: 42.8, 44.9), the standard deviation (SD) was 19 years, and the age ranged from 1 to 100 years. Among the study participants, 777 (61.9%) were males, and 478 were females (38.1%), with a male-to-female ratio of 1.62:1. The gender distribution of the study participants is depicted in Figure 2. A binomial test indicated that the percentage of female patients undergoing colonoscopy of 38.1% was significantly lower than the expected 51.1% with *P* value less than 0.001.

### Indications for colonoscopy

**Figure 3:**
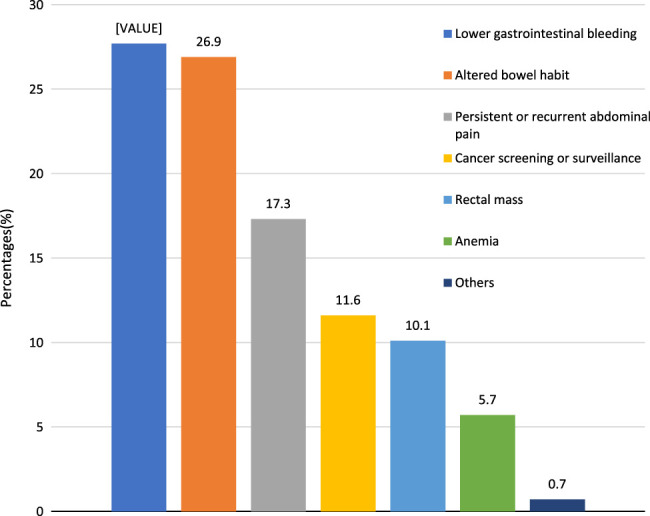
Indications for colonoscopy in the study participants (*n*=1255).

Common indications for colonoscopy included lower gastrointestinal bleeding (*n*=348, 27.7%), altered bowel habit (*n*=337, 26.9%), persistent or recurrent abdominal pain (*n*=217, 17.3%), cancer screening or surveillance (*n*=145, 11.6%), rectal mass (*n*=127, 10.1%) and anaemia (*n*=71, 5.7%). Other indications were thickened bowel walls noted during abdominal imaging (*n*=6), foreign bodies (*n*=2), rectovaginal fistula (*n*=1) and colo-uterine fistula (*n*=1). Reported indications are illustrated in Figure 3.

### Findings of colonoscopy

No abnormalities were detected on colonoscopy in 535 cases (42.6%). Hence, the overall diagnostic yield of colonoscopy was (57.5%). Values for diagnostic yield according to the various indications for colonoscopy are listed in Table 1.

**Table 1:** Diagnostic yield of colonoscopy for various indications

Indication	Significant finding present	Total no. cases	Diagnostic yield (%)
Rectal mass	102	127	80.3
Lower gastrointestinal bleeding	239	348	68.7
Anaemia	42	71	59.2
Persistent or recurrent abdominal pain	115	217	53
Cancer screening or surveillance	69	145	47.6
Altered bowel habit	146	337	43.3

**Figure 4:**
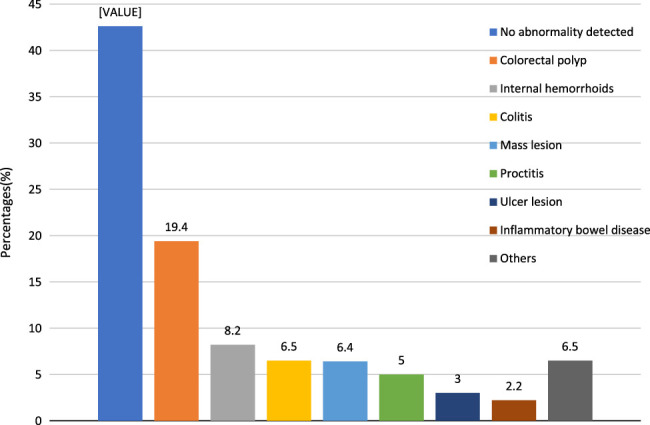
Findings of colonoscopy in the study participants (*n*=1255).

Common findings of colonoscopy included colorectal polyp (*n*=244, 19.4%), internal haemorrhoids (*n*=103, 8.2%), colitis (*n*=82, 6.5%), mass lesion (*n*=80, 6.4%), proctitis (*n*=63, 5%), ulcer lesion (*n*=38, 3%) and inflammatory bowel disease (*n*=28, 2.2%). Other findings included terminal ileitis (*n*=21), diverticulosis (*n*=16), stenosis or stricture (*n*=12), worm infestation (*n*=10), angiodysplasia (*n*=8), blood clots (*n*=4), pseudo-melanosis coli (*n*=4), peri-appendiceal inflammation (*n*=3), foreign body (*n*=2), dilated caecal veins (*n*=1) and rectovaginal fistula (*n*=1). We found the polyp detection rate (PDR) to be 19.4%. The reported findings are illustrated in Figure 4.

### Histopathological findings

Among the study participants, histopathological examination was done in 26.3% (*n*=330) of the cases. Among these 330 cases, no histopathological abnormalities were detected in 34 cases. Common abnormal findings included colitis (*n*=39), hyperplastic polyp (*n*=37), adenocarcinoma (*n*=32), juvenile polyp (*n*=30) and adenoma (*n*=23) as shown in Table 2. Records of histopathological examination couldn’t be retrieved in 63 cases.

**Table 2:** Histopathological findings in patients who underwent biopsy (*n*=330)

Histopathological finding	Frequency (Percentage), *n* (%)
Neoplastic lesions	
Adenocarcinoma	32 (9.7)
Adenoma	23 (7.0)
Other neoplasms	12 (3.6)
Non-neoplastic lesions	
Hyperplastic polyp	37 (11.2)
Juvenile polyp	30 (9.1)
Hamartomatous polyp	1 (0.3)
Inflammatory polyp	11 (3.3)
Colonic lipoma	2 (0.6)
Inflammatory lesions	
Colitis	39 (11.8)
Ileitis	4 (1.2)
Proctitis	5 (1.5)
Granulomatous colitis	17 (5.2)
Tuberculosis	6 (1.8)
Crohn’s disease	5 (1.5)
Ulcerative colitis	9 (2.7)
No abnormality detected	34 (10.3)
Records not retrievable	63 (19.1)
Total	330 (100)

### Association between demographic parameters and presence of colorectal adenocarcinoma

Pearson’s χ^2^ test of independence was performed, which demonstrated that among patients who underwent colonoscopy, having an age of 50 or more years was significantly associated with the presence of colorectal adenocarcinoma (*P* <0.05) with an odds ratio of 2.35 (95% CI: 1.14, 4.89).

Furthermore, having a female sex was found to be significantly associated with the presence of colorectal adenocarcinoma (*P* <0.05, χ^2^ test) with an odds ratio of 2.43 (95% CI: 1.19, 4.97).

Details of the Pearson’s χ^2^ test of independence are listed in Table 3.

**Table 3:** Association between demographic parameters and presence of colorectal adenocarcinoma

Variables	Adenocarcinoma present, *n* (%)	Adenocarcinoma absent, *n* (%)	Total (*n*)	χ^2^ statistic	Degrees of freedom	*P*
Age						
≥50 years	20 (3.8)	507 (96.2)	527	5.670	1	0.017
<50 years	12 (1.6)	716 (98.4)	728			
Sex						
Female	19 (4)	459 (96)	478	6.310	1	0.012
Male	13 (1.7)	764 (98.3)	777			

## Discussion

We found that the mean age of the patients who underwent colonoscopy was 43.8 years, which is comparable to other similar studies done in Nepal^5–7^. However, the median age of patients undergoing colonoscopy was reported to be 62.5 years in a study done in the USA, 57 years in a general hospital in the UK and 59 years in a study done in South Korea^10–12^. In Nepal, the use of colonoscopy as a screening tool for colorectal carcinoma (CRC) in older individuals has not been widely established yet. This could be one of the reasons why the average age of patients undergoing colonoscopy is lower in Nepal compared to developed countries.

The majority of the patients who underwent colonoscopy were males. The observed male-to-female ratio was 1.62:1. Studies have identified that lower socioeconomic status and lower education status among women could be responsible for the lower healthcare accessibility among women when compared to men in Nepal^13^. This gender disparity could be responsible for the increased male-to-female ratio among patients who utilized colonoscopy services at our centre.

In our study, only 11.6% of the colonoscopies were done as a screening or surveillance tool for CRC. In developed countries, a larger proportion of the colonoscopies are performed as a screening and surveillance tool for CRC in older individuals^10,11^. The American College of Gastroenterology (ACG) strongly recommends using colonoscopy as a screening tool for CRC in individuals between 50 and 75^14^. In Nepal, such guidelines have not been formulated yet. Hence, the use of colonoscopy as a screening tool for CRC has not been widely established.

The American Society for Gastrointestinal Endoscopy (ASGE)/American College of Gastroenterology (ACG) Task Force on Quality in Endoscopy recommended that less than 15% of total colonoscopies performed should have inadequate bowel preparation^15^. At our centre, only 3% of the patients who underwent colonoscopy were found to have inadequate bowel preparation. During the preparation of our patients for colonoscopy, trained staff provide easy-to-follow and effective instructions in the native language with frequent reminders and the use of pamphlets. This contributed to reducing the proportion of patients with inadequate bowel preparation.

The overall diagnostic yield in our study was 57.5%, with the highest yield in cases for suspected rectal mass (80.3%) followed by lower gastrointestinal bleeding (68.7%). Thus, probability of detecting a lesion on colonoscopy was highest in patients with rectal mass that was identified during a rectal examination or proctoscopy. The diagnostic yield was comparatively low (43.3%) when colonoscopy was performed in patients with an altered bowel habit. At our centre, in all patients with an altered bowel habit suspected to be suffering from irritable bowel syndrome (IBS), we perform a colonoscopy to rule out inflammatory bowel disease (IBD). The American College of Gastroenterology (ACG) recommends testing for faecal calprotectin to rule out IBD in cases of suspected IBS^16^. However, due to the unavailability of any tests for faecal calprotectin at our centre, we use colonoscopy for the same purpose. This could have contributed to the low diagnostic yield in patients with an altered bowel habit.

Adenoma detection rate (ADR) is the fraction of patients undergoing colonoscopy who had one or more adenomas detected^15^. ADR is widely used as an indicator of quality in colonoscopies. The ASGE/ACG Task Force on Quality in Endoscopy recommends ADR targets of greater than 25%. The PDR is the number of patients with at least one polyp removed during colonoscopy. PDR has been found to correlate well with ADR by several studies^15^. At our centre, we found the PDR to be 19.4%.

CRC was previously reported to be less prevalent in Asians than in the western population^17^. This was attributed to differences in dietary habits and lifestyle. However, recent studies have shown that incidence rates of CRC in Asian countries are increasing and approaching the incidences in western countries. This could have been caused by changes in diet and lifestyle in the Asian population^17^. In Nepal, the annual incidence rate of CRC in the older population (45–75-year-olds) is increasing at 6.7% per year. This is in contrast to western countries where the incidence rate in the older population is decreasing due to effective screening colonoscopies for CRC^18^. Despite such a growing incidence of CRC, there is the paucity of studies exploring the risk factors of CRC in the Nepalese population. Furthermore, guidelines for screening and surveillance of CRC have not been implemented in the healthcare delivery system of Nepal^18,19^.

In our study, patients who underwent colonoscopy at the age of 50 or more were significantly associated with the presence of colorectal adenocarcinoma. This is in accordance with other studies done previously in Nepal and worldwide^18,20^. Furthermore, we also report that at our centre, having a female sex was found to be significantly associated with the presence of colorectal adenocarcinoma. This is in contrast to global studies on colorectal cancer, which report higher prevalence in males when compared to females^20^. This discrepancy could have been caused by differences in genetics, diet and lifestyle^17^.

We understand that since this study was carried out with records of only those patients who underwent colonoscopy, the results may not be representative of the entire population. We also acknowledge that since this study was carried out at a single centre, it might not be representative of all colonoscopies carried out in Nepal. However, we believe this study provides valuable insights into the pattern of lower gastrointestinal diseases in the Nepalese population.

## Conclusion

In our study, common indications for colonoscopy were lower gastrointestinal bleeding, altered bowel habits and persistent abdominal pain. Compared to studies in developed countries, screening colonoscopies comprised a smaller fraction.

Furthermore, common findings during the colonoscopy included colorectal polyps, internal haemorrhoids and colitis. Among patients who underwent colonoscopy, being of female sex as well as having an age of 50 or more years were both significantly associated with the presence of colorectal adenocarcinoma. The gender predisposition of CRC in our study is in contrast with the global trend.

The incidence of CRC is growing in Nepal. However, there is the paucity of studies concerning the risk factors and need for screening of CRC in Nepal. We recommend the conduction of multicenter, prospective studies to explore the sex-specific as well as general risk factors of CRC in the Nepalese population. Studies aimed at understanding barriers preventing women from accessing colonoscopy services are warranted. The need of CRC screening should be addressed in the healthcare delivery system of Nepal.

## Ethical approval

This study was done in Dhulikhel Hospital, Kathmandu University School of Medical Sciences. Ethical approval was obtained from the Institutional Review Committee of Dhulikhel Hospital, Kathmandu University School of Medical Sciences (KUSMS-IRC) with approval number: 119/20.

## Consent

This was a retrospective conducted on de-identified data from hospital records. Informed consent was not obtained from each individual patient in line with the recommendation of our Ethical Committee. All patients were able to refuse/withdraw the use of data according to the hospital policy and information to the patients.

## Source of funding

This work did not receive any funding. There are no sources of funding to be declared.

## Author contribution

P. Sharma, P. Sapkota and R.B.G. contributed to patient care, conceptualization, design and reviewing. P. Silwal, B.Y. and N.G. contributed to contributed to design, writing the original draft, reviewing and editing. S. Pathak, S.J. and Y.S. contributed to writing the original draft, reviewing and editing.

## Conflicts of interest disclosure

The authors declare that they have no financial or personal conflicts of interest with regard to the content of this publication.

## Research registration unique identifying number (UIN)

Name of registry: Open Science Framework (OSF) registry Link to Registration: https://doi.org/10.17605/OSF.IO/TG395 Unique identifying number: osf.io/tg395.

## Guarantor

Pasanda Sharma.

## Data availability statement

All data that support the findings of this study can be obtained by contacting the authors.

## Provenance and peer review

Not commissioned, externally peer-reviewed.

## References

[1] Naseer O Bashir Rishi M Gelia AM. Clinical characteristics and main findings of colonoscopy in Tripoli Central Hospital: a cross-sectional study of 1858 patients. Cureus 2023;15:e34983. 36938214 10.7759/cureus.34983 PMC10019830

[2] Ashtari S. Overview of diagnostic and treatment colonoscopy function in gastrointestinal diseases. J Liver Res Disord Ther 2016;2:00035.

[3] Waye JD. Colonoscopy. CA Cancer J Clin 1992;42:350–365. 1393743 10.3322/canjclin.42.6.350

[4] Bhattarai S Acharya RR. Clinical profile and endoscopic findings in patients undergoing colonoscopy at a tertiary care centre of western Nepal. J Coll Med Sci-Nepal 2020;16:66–70.

[5] Belbase NP Karki M Dewan KR. Demographic profile of patients undergoing colonoscopy at a tertiary care centre in central Nepal. J Coll Med Sci-Nepal 2021;17:16–22.

[6] Koirala D Pathak R Kafle Bhandari B. Detection of Colonic polyps during colonoscopy in a tertiary care center of Nepal. J Nepal Health Res Counc 2021;19:596–602. 35140437 10.33314/jnhrc.v19i3.3678

[7] Chaudhary S Chaudhary P Jaiswal N. Colonoscopy: a two year experience from western Nepal. J Univ Coll Med Sci 2013;1:28–32.

[8] Mathew G Agha R Albrecht J. STROCSS 2021: Strengthening the reporting of cohort, cross-sectional and case-control studies in surgery. Int J Surg 2021;96:106165. 34774726 10.1016/j.ijsu.2021.106165

[9] Pourhoseingholi MA Vahedi M Rahimzadeh M. Sample size calculation in medical studies. Gastroenterol Hepatol Bed Bench 2013;6:14–17. 24834239 PMC4017493

[10] Thomas-Gibson S Thapar C Shah SG. Colonoscopy at a Combined District General Hospital and Specialist Endoscopy Unit: Lessons from 505 Consecutive Examinations. *J R Soc Med*. Published online April 1, 2002 doi:10.1177/014107680209500408 PMC1279514 11934910

[11] Boroff ES Disbrow M Crowell MD. Adenoma and polyp detection rates in colonoscopy according to indication. Gastroenterol Res Pract 2017;2017:7207595. 29445393 10.1155/2017/7207595 PMC5763113

[12] Lee JG Han DS Joo YE. Colonoscopy quality in community hospitals and nonhospital facilities in Korea. Korean J Intern Med 2020;36(suppl 1):S35–S43. 32388944 10.3904/kjim.2019.117 PMC8009161

[13] Ashworth HC Roux TL Buggy CJ. Healthcare accessibility in the rural plains (terai) of Nepal: physical factors and associated attitudes of the local population. Int Health 2019;11:528–535. 30916330 10.1093/inthealth/ihz008

[14] Shaukat A Kahi CJ Burke CA. ACG Clinical Guidelines: Colorectal Cancer Screening 2021. Am J Gastroenterol 2021;116:458–479. 33657038 10.14309/ajg.0000000000001122

[15] Rex DK Schoenfeld PS Cohen J. Quality indicators for colonoscopy. Gastrointest Endosc 2015;81:31–53. 25480100 10.1016/j.gie.2014.07.058

[16] Lacy BE Pimentel M Brenner DM. ACG Clinical Guideline: Management of Irritable Bowel Syndrome. Am J Gastroenterol 2021;116:17–44. 33315591 10.14309/ajg.0000000000001036

[17] Pardamean CI Sudigyo D Budiarto A. Changing colorectal cancer trends in Asians: epidemiology and risk factors. Oncol Rev 2023;17:10576. 37284188 10.3389/or.2023.10576 PMC10241074

[18] Shrestha G Khanal S Mulmi R. Five-year trend of colorectal cancer incidence in B.P. Koirala Memorial Cancer Hospital of Central Nepal: a cross-sectional study. Int J Surg Glob Health 2020;3:e30–e30.

[19] Kumar A Dhungana SB Gupta RK. Clinico-pathological characteristics of obstructing colorectal cancer and its management outcomes at a tertiary referral center of Eastern Nepal. BMC Gastroenterol 2022;22:1–6. 35729511 10.1186/s12876-022-02380-0 PMC9210611

[20] Wong MCS Huang J Huang JLW. Global prevalence of colorectal neoplasia: a systematic review and meta-analysis. Clin Gastroenterol Hepatol 2020;18:553–561.e10. 31323383 10.1016/j.cgh.2019.07.016

